# DYRK1A suppression restrains Mcl-1 expression and sensitizes NSCLC cells to Bcl-2 inhibitors

**DOI:** 10.20892/j.issn.2095-3941.2019.0380

**Published:** 2020-05-15

**Authors:** Yangling Li, Dongmei Zhou, Shuang Xu, Mingjun Rao, Zuoyan Zhang, Linwen Wu, Chong Zhang, Nengming Lin

**Affiliations:** ^1^Department of Clinical Pharmacology, Hangzhou First People’s Hospital, Nanjing Medical University, Hangzhou 310006, China; ^2^Department of Clinical Pharmacology, Key Laboratory of Clinical Cancer Pharmacology and Toxicology Research of Zhejiang Province, Hangzhou First People’s Hospital, Zhejiang University School of Medicine, Hangzhou 310006, China; ^3^Institute of Pharmacology, College of Pharmaceutical Sciences, Zhejiang Chinese Medical University, Hangzhou 311402, China; ^4^School of Medicine, Zhejiang University City College, Hangzhou 310015, China

**Keywords:** DYRK1A, Mcl-1, NSCLC, combination, Bcl-2 inhibitor

## Abstract

**Objective:** Mcl-1 overexpression confers acquired resistance to Bcl-2 inhibitors in non-small cell lung cancer (NSCLC), but no direct Mcl-1 inhibitor is currently available for clinical use. Thus, novel therapeutic strategies are urgently needed to target Mcl-1 and sensitize the anti-NSCLC activity of Bcl-2 inhibitors.

**Methods:** Cell proliferation was measured using sulforhodamine B and colony formation assays, and apoptosis was detected with Annexin V-FITC staining. Gene expression was manipulated using siRNAs and plasmids. Real-time PCR and Western blot were used to measure mRNA and protein levels. Immunoprecipitation and immunofluorescence were used to analyze co-localization of dual specificity tyrosine-phosphorylation-regulated kinase 1A (DYRK1A) and Mcl-1.

**Results:** Suppression of DYRK1A resulted in reduced Mcl-1 expression in NSCLC cells, whereas overexpression of DYRK1A significantly increased Mcl-1 expression. Suppression of DYRK1A did not alter Mcl-1 mRNA levels, but did result in an accelerated degradation of Mcl-1 protein in NSCLC cells. Furthermore, DYRK1A mediated proteasome-dependent degradation of Mcl-1 in NSCLC cells, and DYRK1A co-localized with Mcl-1 in NSCLC cells and was co-expressed with Mcl-1 in tumor samples from lung cancer patients, suggesting that Mcl-1 may be a novel DYRK1A substrate. We showed that combined therapy with harmine and Bcl-2 antagonists significantly inhibited cell proliferation and induced apoptosis in NSCLC cell lines as well as primary NSCLC cells.

**Conclusions:** Mcl-1 is a novel DYRK1A substrate, and the role of DYRK1A in promoting Mcl-1 stability makes it an attractive target for decreasing Bcl-2 inhibitor resistance.

## Introduction

The B-cell lymphoma-2 (Bcl-2) family of proteins are characterized by four homology domains, BH1-BH4, and are subdivided into two major groups. The first of these is the anti-apoptotic subgroup, which includes Bcl-2 and Mcl-1. The second is the proapoptotic subgroup, which includes Bax-like molecules (such as Bax and Bak) that contain the BH1-3 domains, as well as BH3-only proteins (such as Bid and Bad)^1^. When BH3-only proteins are activated, their amphipathic α-helical BH3 domain inserts into a hydrophobic groove on Bcl-2 anti-apoptotic proteins, subsequently initiating apoptosis^2^. Small molecule Bcl-2 inhibitors are promising for the treatment of hematological malignancies in addition to other cancers^3^. ABT-737 (Navitoclax), the first BH3 mimetic, has shown promising effects in clinical trials for patients with relapsed chronic lymphocytic leukemia (CLL)^4^. Consequently, ABT-199 (Venetoclax) became the first BH3 mimetic approved for cancer treatment by the Food and Drug Administration (FDA) on April 11, 2016^5^. ABT-199 is an oral Bcl-2 specific inhibitor and is approved to treat CLL patients with the 17p deletion who have received at least one prior therapy^6,7^. Because Bcl-2 inhibitors bind to Mcl-1 with low affinity, Mcl-1 overexpression confers acquired resistance to Bcl-2 inhibitors in multiple cancer types, including non-small cell lung cancer (NSCLC) and acute myeloid leukemia^8,9^. NSCLC is the most common malignancy worldwide and accounts for ~85% of all lung cancers, with small cell lung cancer comprising ∼15%^10^. In addition, Mcl-1 is a novel biomarker of poor prognoses for NSCLC patients^11^. However, there is no direct Mcl-1 inhibitor currently available in clinical use. Thus, novel therapeutic strategies are urgently needed to target Mcl-1 and sensitize the anti-cancer activities of Bcl-2 inhibitors for effective treatment of NSCLC.

DYRK1A is located on the critical region of Down syndrome (DS) chromosome 21, and its overexpression in DS patients contributes to cognitive impairments^12^. In addition, DYRK1A is thought to be involved in neurodegenerative diseases including Alzheimer’s disease, Parkinson’s disease, and Huntington’s disease^13^. DYRK1A is also overexpressed in multiple human malignancies, including hematological and brain cancers, where it contributes to tumor growth by manipulating cell cycle progression^14–16^. Thus, its role in multiple human diseases makes DYRK1A an attractive target for therapeutic drugs^17^. Harmine, a β-carboline alkaloid, is a selective ATP-competitive inhibitor of DYRK1A, which acts by binding to the ATP-binding pocket of DYRK1A^18^. Inhibition of DYRK1A with harmine reverses resistance to multiple anti-cancer drugs including mitoxantrone, camptothecin, and AZD9291^19,20^.

DYRK1A is a serine/threonine kinase that regulates diverse pathways involved in cell differentiation and apoptosis, gene transcription and splicing, and the cell cycle^21^. DYRK1A phosphorylates NFATc1, p27, EGFR, and c-myc at serine or threonine residues^22^. Here, we showed that Mcl-1 was a novel DYRK1A substrate, and DYRK1A suppression sensitized NSCLC cells to Bcl-2 inhibitors.

## Materials and methods

### Materials

DMEM/F-12, RPMI-1640, and fetal bovine serum (FBS) were obtained from Gibco (Grand Island, NY, USA). Harmine was provided by MedChemExpress (HY-N0737A; Monmouth Junction, NJ, USA). ABT-737 (S1002) and ABT-199 (S8048) were provided by Selleck Chemicals (Houston, TX, USA). The Annexin V-FITC apoptosis kit was provided by Becton Dickinson (556547; Franklin Lakes, NJ, USA). The primary antibody against glyceraldehyde 3-phosphate dehydrogenase (GAPDH) was provided by Santa Cruz Biotechnology (sc-32233; Santa Cruz, CA, USA). The primary antibodies against Bcl-2 (ab32124) and Bcl-xL (ab32370) were provided by Abcam (Cambridge, MA, USA). The primary antibodies against Mcl-1 (94296), α/β-Tubulin (2148S), caspase-3 (9662S), phospho-Mcl-1 (Ser159/Thr163; 4579S), DYRK1A (2771S), and cleaved-PARP (9541S) were provided by Cell Signaling Technology (Danvers, MA, USA). The horseradish peroxidase-labeled secondary anti-mouse (AS003) and anti-rabbit (AS014) antibodies were provided by ABclonal Technology (Woburn, MA, USA). The primary antibodies were diluted with Primary Antibody Dilution Buffer (P0023A; Beyotime, Shanghai, China) at a ratio of 1:1,000.

### Isolation of primary tumor cells from NSCLC patients

Tumor samples were collected from NSCLC patients and informed consent was obtained from all participants. All patient-related research was approved by the Clinical Ethics Committee of Hangzhou First People’s Hospital before study initiation (Approval No. 2016/21-01)^20^. The research was conducted according to the World Medical Association Declaration of Helsinki.

### Cell culture

Human lung carcinoma cell lines (A549, NCI-H1299, and NCI-H460) were provided by Shanghai Institute of Biochemistry and Cell Biology (Shanghai, China). A549, NCI-H460, and NCI-H1299 cells were cultured in RPMI-1640 media with 10% FBS, and primary NSCLC cells were cultured in DMEM/F-12 with 10% FBS. The cells were maintained in humidified conditions (37 °C, 5% CO_2_).

### The sulforhodamine B (SRB) assay

Cell viability was measured using the SRB assay. Cells (5 × 10^3^) were seeded on 96-well plates and treated with compounds for 72 h. The cells were fixed with 10% (w/v) trichloroacetic acid overnight, washed 5 times with deionized water, and dried at 60 °C. After the plates were entirely dry, cells were stained using 100 μL 0.4% SRB solution for 30 min, washed using 1% (v/v) acetic acid, and dried at 60 °C. Protein-bound dye was dissolved with a Tris-based solution and cell viability was measured at 540 nm using a multi-scan spectrum.

### Colony formation assay

NSCLC cells were cultured on 6-well plates (1,000 cells per well) overnight, incubated with the compounds for 14 days, fixed with 4% paraformaldehyde for 15 min, and stained using Giemsa solution for another 15 min. Colonies were photographed using ChemiDoc XPS (Bio-Rad, Hercules, CA, USA).

### Apoptosis assay

Cells were collected, washed with phosphate-buffered saline (PBS), stained using the Annexin V-FITC Apoptosis Kit (BD Biosciences, Franklin Lakes, NJ, USA), and detected using flow cytometry. Briefly, 1 × 10^5^ cells were harvested and resuspend in 200 μL of Annexin V binding buffer containing 5 μL of Annexin V-FITC for 15 min at room temp in the dark. And 5 μL of 0.5 mg/mL propidium iodide solution was added before flow cytometer detection.

### Silencing of gene expression with small interfering RNA (siRNA)

The cells were seeded on 6-well plates, cultured overnight, and transfected the next day with DYRK1A siRNA using Oligofectamine (Invitrogen, Waltham, MA, USA). Twenty nM siRNA/well was used in a 6-well plate with 100 μL jetPRIME buffer and 2 μL jetPRIME. After 48 h of incubation, the cells were harvested for further analysis. The efficiency of transfection was assessed by Western blot. DYRK1A siRNA was obtained from GenePharma (Shanghai, China), and the sense sequences were as follows: siDYRK1A-1: 5′-AUGGAGCUAUGGACGUUAATT-3′, siDYRK1A-2: 5′-AAACUCGAAUUCAACCUUATT-3′, negative control: 5′-UUCUCCGAACGUGUCACGUTT-3′.

### Real-time reverse transcription PCR

Total cellular RNA was extracted with TRIzol (Invitrogen, Carlsbad, CA, USA), precipitated with isopropyl alcohol, and rinsed with 70% ethanol. Single stranded cDNA was generated using oligo (dT) priming, followed by SYBR Green-based real-time reverse transcription PCR (Bio-Rad Laboratories, Hercules, CA, USA). After the primers were received, they were centrifuged and then diluted to 10 μM as stock solutions. SYBR Green qPCR Master Mix Kit was used to establish a 10 μL reaction system [cDNA: 1.0 μL, primer-forward: 1.0 μL, primer-reverse: 1.0 μL, ddH20: 1.8 μL, ROX Reference Dye (50×): 0.2 μL, SYBR Green (2×): 5 μL]. Amplification conditions were 95 °C for 15 min, followed by 40 thermal cycles of 15 s at 95 °C, 30 s at 58 °C, and 30 s at 72 °C. A standard curve-based method was used to measure the expression of the indicated mRNA. The primers used in the present study were as follows: DYRK1A, 5′-TCTGGGTATTCCACCTGCTC-3′ (forward), 5′-GTCCTCCTGTTTCCACTCCA-3′ (reverse); Mcl-1, 5′-GGGCAGGATTGTGACTCTCATT-3′ (forward), 5′-GATGCAGCTTTCTTGGTTTATGG-3′ (reverse); GAPDH, 5′-GAGTCAACGGATTTGGTCGT-3′ (forward), 5′-TTGATTTTGGAGGGATCTCG-3′ (reverse).

### Plasmid transfection

NSCLC cells were cultured on 6-well plates overnight, and transfection was performed the next day using jetPRIME (Polyplus, Illkirck, France) according to the manufacturer’s instructions. Briefly, 1 μg plasmid was diluted into 100 μL jetPRIME^®^ buffer. Then, 2 μL jetPRIME^®^ was added, vortexed, and incubated for 10 min at room temperature. Transfection mix (100 μL/well) was added to the cells.

### Western blot analysis

Cells were lysed using RIPA Buffer (Thermo Fisher Scientific, Waltham, MA, USA). Total protein (20 μg) was subjected to 10% SDS-PAGE and transferred to a polyvinylidene difluoride membrane. The membranes were blocked and then incubated with primary and secondary antibodies. Protein expression was analyzed using an enhanced chemiluminescence (ECL, Abbkine, Wuhan, China) system.

### Immunoprecipitation

Cell lysates were centrifuged at 10,000 × *g* for 30 min at 4 °C, incubated with primary antibody using a slow rotation for 4 h, and then incubated with protein G magnetic beads for 1 h at 4 °C. The beads were washed a minimum of five times, mixed with loading buffer, heated to 95 °C for 5 min, followed by Western blot analysis.

### Immunofluorescence

Cells were seeded into 96-well plates and cultured overnight. The next day, the cells were fixed with 4% paraformaldehyde for 30 min at room temperature, washed with PBS, and permeabilized with 200 μL of 0.3% Triton X-100 in PBS for 30 min. Cells were washed again with PBS, blocked with 1% bovine serum albumin in PBS for 30 min, incubated with primary antibodies at 4 °C overnight, washed three times with PBS, and incubated with fluorescent dye-conjugated secondary antibodies for 1 h in the dark. Nuclei were stained with 4′,6-diamidino-2-phenylindole for 5 min in the dark. Cells were visualized and photographed using a fluorescence microscope.

### Statistical analysis

The results are expressed as the mean ± SD. The data presented were obtained at least three times. The synergistic effects of harmine plus ABT-199/ABT-737 were quantitatively determined by calculating the combination index (CI) values using Calcusyn software. A CI value < 0.9 indicated synergism; 0.9–1.1, additive; > 1.1, antagonism. A two-tailed Student’s *t*-test was used to compare the difference between two groups. A value of *P* < 0.05 was accepted as statistically significant, and the levels of significance were indicated as follows: ^*^P < 0.05, ^**^P < 0.01, and ^***^P < 0.001.

## Results

### DYRK1A upregulates Mcl-1 expression in NSCLC cells

**Figure 1:**
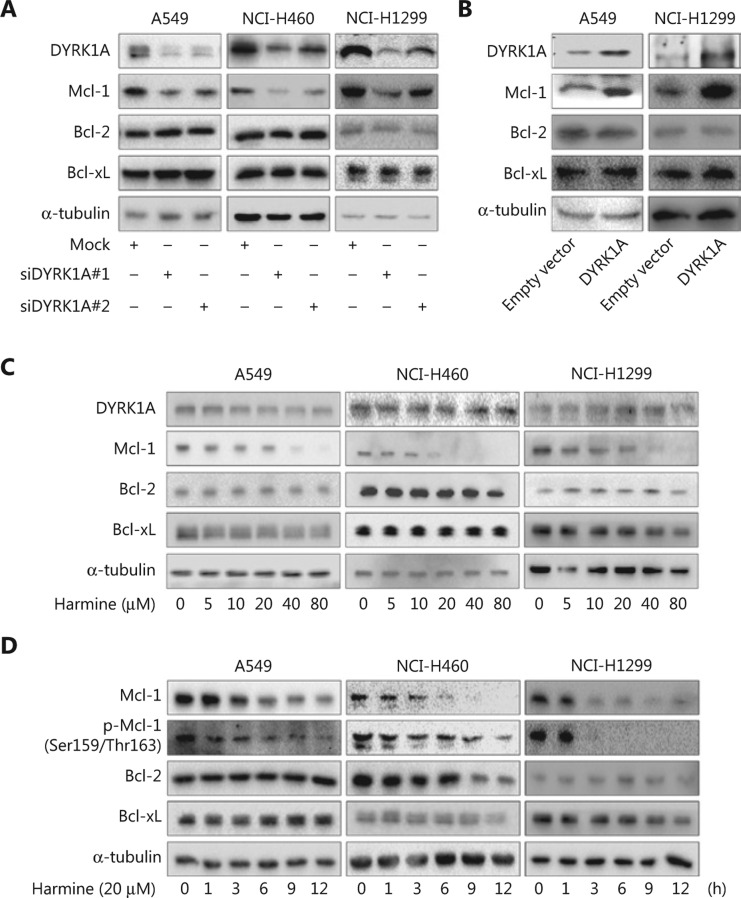
DYRK1A regulates the expression of Mcl-1 in NSCLC cells. (A) NSCLC cells were transfected with control siRNA and DYRK1A siRNA for 48 h, and the expression of DYRK1A and Bcl-2 family members were detected by Western blot. (B) NSCLC cells were transfected with empty vector or DYRK1A plasmid for 48 h, and the expression of DYRK1A and Bcl-2 family members were detected by Western blot. (C) NSCLC cells were treated with harmine at the indicated concentrations for 24 h, and the expression of the indicated proteins were detected by Western blot. (D) NSCLC cells were treated with 20 μM harmine for 1, 3, 6, 9, and 12 h, and the expression of the indicated proteins was detected by Western blot.

DYRK1A expression was knocked down in NSCLC cell lines using siRNA, and DYRK1A knockdown resulted in decreased Mcl-1 expression in NSCLC cells (**Figure 1A**). In contrast, overexpression of DYRK1A in NSCLC cells significantly increased Mcl-1expression (**Figure 1B**). Expression of other Bcl-2 family members, such as Bcl-2 and Bcl-xL, was not altered with DYRK1A knockdown or overexpression in NSCLC cells (**Figure 1A and 1B**). Treatment of NSCLC cells with harmine, a DYRK1A inhibitor, resulted in a dose- and time-dependent inhibition of Mcl-1 expression (**Figure 1C and 1D**). However, Bcl-2 and Bcl-xl expression was not changed when DYRK1A was inhibited with harmine in NSCLC cells (**Figure 1C and 1D**). These data suggested that DYRK1A upregulated Mcl-1 expression in NSCLC cells.

### DYRK1A promotes the stability of Mcl-1 in NSCLC cells

**Figure 2:**
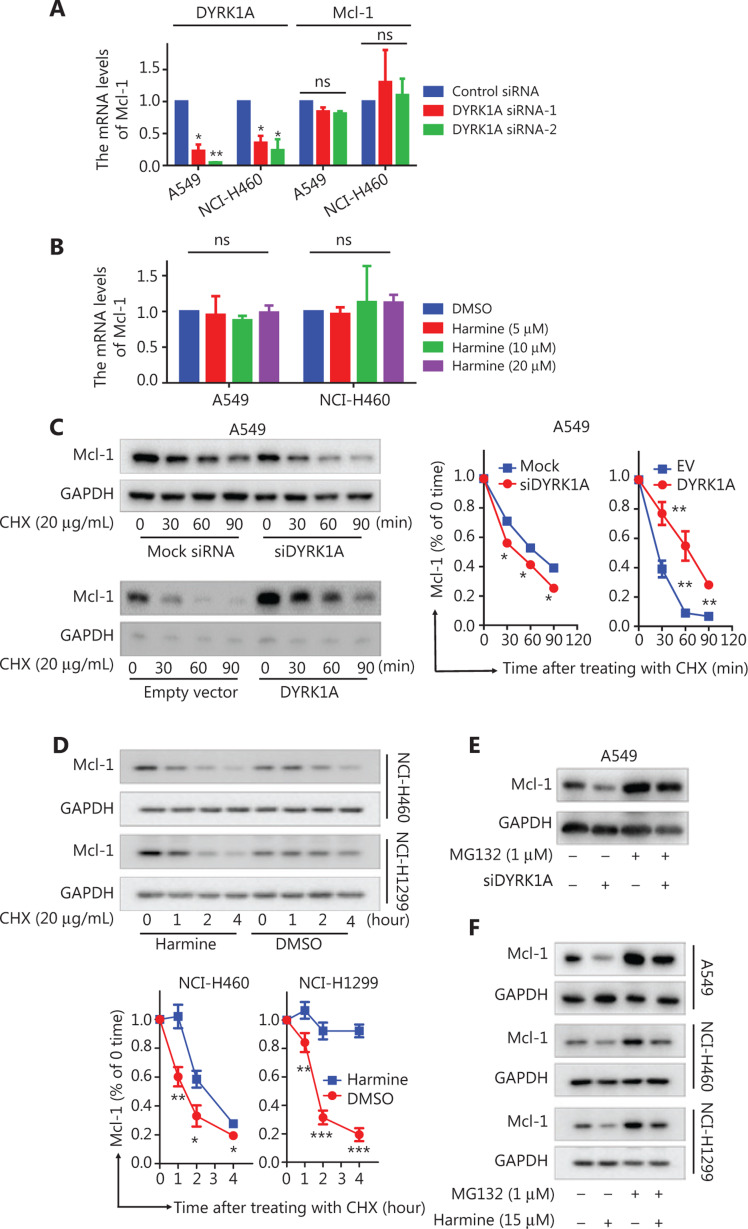
DYRK1A promotes the stability of Mcl-1 in NSCLC cells. (A) NSCLC cells were transfected with control siRNA and DYRK1A siRNA for 48 h, and real-time reverse transcriptase PCR analyses were used to detect the mRNA levels of DYRK1A and Mcl-1. (B) NSCLC cells were treated with harmine for 24 h, and real-time reverse transcriptase PCR analyses were used to detect the mRNA levels of Mcl-1. (C) A549 cells were transfected with siDYRK1A or DYRK1A plasmid for 48 h. Then, cells were treated with cycloheximide (CHX; 20 μg/mL) to block new protein synthesis, and the degradation of Mcl-1 was detected by Western blot. (D) NSCLC cells were treated with CHX (20 μg/mL) in the absence or presence of 15 μM harmine for 1, 2, 3, and 4 h, after which Western blot analysis was performed to measure Mcl-1 levels. (E) A549 cells were transfected with siDYRK1A or mock siRNA for 48 h, and cells were exposed to MG132 (1 μM) or dimethylsulfoxide (DMSO) for 24 h, then Western blot analysis was performed to measure Mcl-1 levels. (F) NSCLC cells were treated with MG132 (1 μM) and/or harmine (15 μM) for 24 h, then Western blot analysis was performed to measure Mcl-1 levels. Significant results are presented as ns non-significant, * *P* < 0.05, ** *P* < 0.01, or *** *P* < 0.001.

To investigate whether DYRK1A regulates Mcl-1 protein expression at the transcriptional level, Mcl-1 mRNA expression was measured in NSCLC cells after treatment with siDYRK1A or harmine. After treatment with siRNA, depletion of DYRK1A did not inhibit Mcl-1 mRNA expression (**Figure 2A**, the Mcl-1 mRNA levels in the DYRK1A siRNA group *vs.* control siRNA group, *P* > 0.05). Likewise, inhibition of DYRK1A with harmine did not change Mcl-1 mRNA expression (**Figure 2B**). Next, we hypothesized that DYRK1A might promote the degradation of Mcl-1 in NSCLC cells. Indeed, depletion of DYRK1A resulted in an accelerated degradation of Mcl-1 protein in A549 cells when new protein synthesis was blocked by cycloheximide (CHX). In contrast, overexpression of DYRK1A in NSCLC cells significantly reduced Mcl-1 protein degradation in NSCLC cells (**Figure 2C**, the Mcl-1 protein levels in DYRK1A overexpression group *vs.* control group, *P* < 0.01). Inhibition of DYRK1A with harmine resulted in accelerated degradation of Mcl-1 in NSCLC cells in the presence of CHX (**Figure 2D**). Furthermore, treatment of cells with the proteasome inhibitor, MG132, prevented the degradation of Mcl-1 that was induced by DYRK1A depletion or inhibition (**Figure 2E and 2F**). These data demonstrated that DYRK1A promoted stability of Mcl-1 in NSCLC cells.

### DYRK1A directly interacts with Mcl-1 in NSCLC cells and is co-expressed with Mcl-1 in tumor samples from lung cancer patients

**Figure 3:**
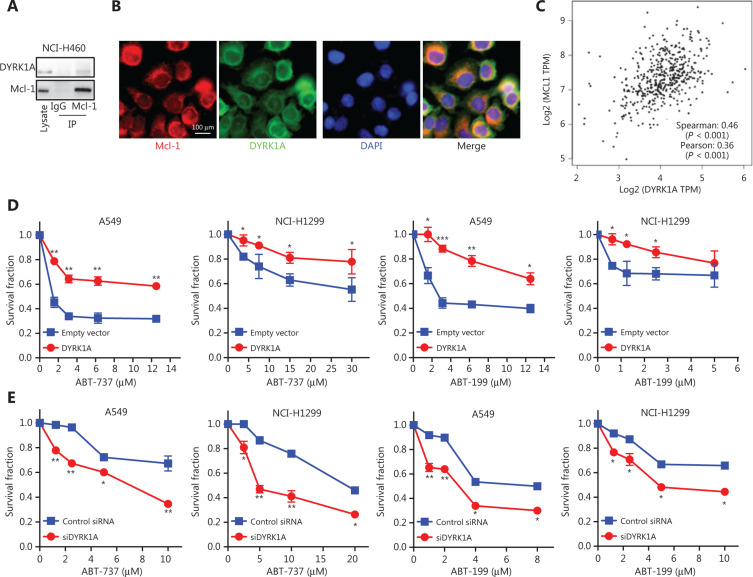
DYRK1A directly interacts with Mcl-1 in NSCLC cells and co-expresses with Mcl-1 in tumor samples from lung cancer patients. (A) The interaction between DYRK1A and Mcl-1 was determined using immunoprecipitation in NCI-H460 cells. (B) The interaction between DYRK1A and Mcl-1 was tested using immunofluorescence in A549 cells. Scale bar: 100 μm. (C) The co-expression data were collected from GEPIA (http://gepia.cancer-pku.cn). Gene: DYRK1A and Mcl-1; Database: LUAD Tumor. (D) NSCLC cells were transfected with empty vector or DYRK1A plasmid for 24 h, seeded on 96-well plates overnight, and treated with Bcl-2 inhibitors (ABT-737 or ABT-263) at indicated concentrations for 72 h. Then, the Sulforhodamine B (SRB) assay was used to evaluate the cell viability. (E) NSCLC cells were transfected with DYRK1A siRNA or mock siRNA for 24 h, seeded on 96-well plates overnight, and treated with Bcl-2 inhibitors (ABT-737 or ABT-263) at indicated concentrations for 72 h. Then, the SRB assay was used to evaluate the cell viability. Significant results are presented as * *P* < 0.05, ** *P* < 0.01, or *** *P* < 0.001.

DYRK1A regulates phosphorylation of many substrates and is involved in controlling a variety of molecular processes^17^. Phosphorylation of Mcl-1 at Thr-163 increases protein stability and is responsible for chemoresistance in NSCLC cells^23^. We found that treatment of cells with harmine significantly reduced Mcl-1 phosphorylation (Thr-163; **Figure 1D**). Thus, we hypothesized that DYRK1A interacted with Mcl-1 to regulate its phosphorylation. Indeed, our data suggested that DYRK1A directly interacted with Mcl-1 in A549 cells, and co-localization of DYRK1A with Mcl-1 was observed in A549 cells using a dual immunofluorescent assay (**Figure 3A and 3B**). Next, co-expression of DYRK1A and Mcl-1 was analyzed in NSCLC patient samples. We found that DYRK1A was co-expressed with Mcl-1 in tumor samples from lung cancer patients (**Figure 3C**)^24^. Mcl-1 overexpression confers acquired resistance to Bcl-2 inhibitors, and therefore we hypothesized that DYRK1A overexpression would also hinder the anti-cancer effects of Bcl-2 inhibitors. Overexpression of DYRK1A by transfecting DYRK1A plasmid decreased the anti-proliferative effects of Bcl-2 inhibitors in both A549 and NCI-H1299 cells (**Figure 3E**). In contrast, suppression of DYRK1A by transfecting with DYRK1A siRNA enhanced the anti-proliferative effects of Bcl-2 inhibitors in NSCLC cells (**Figure 3F**). These data suggested that overexpression of DYRK1A and Mcl-1 promoted acquired resistance to Bcl-2 inhibitors in NSCLC cells.

### DYRK1A inhibition sensitizes NSCLC cells to Bcl-2 inhibitors

**Figure 4:**
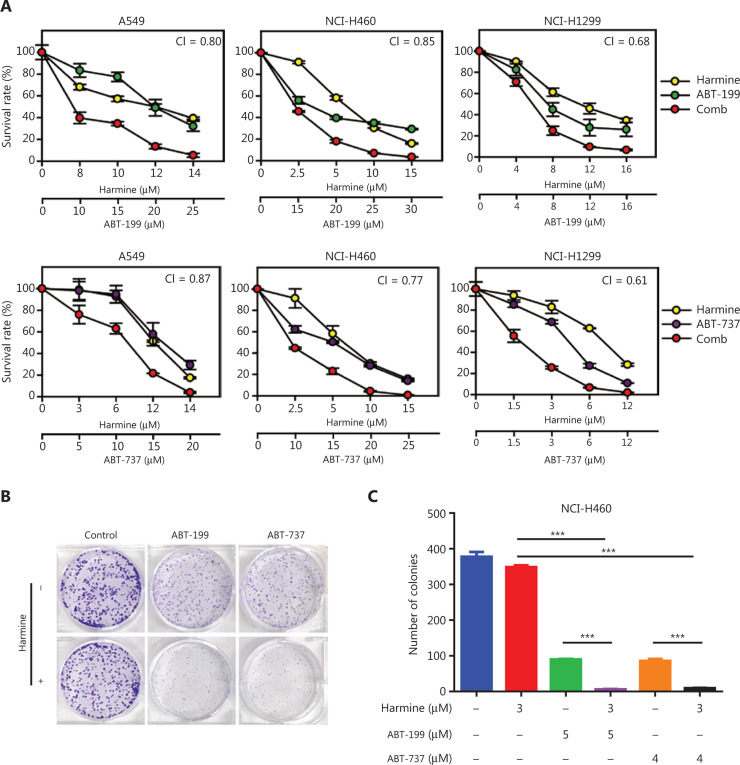
DYRK1A inhibitor, harmine, enhances the anti-proliferation effects of Bcl-2 inhibitors in NSCLC cells. (A) NSCLC cells were treated with DYRK1A inhibitor harmine and/or Bcl-2 inhibitor (ABT-737 or ABT-263) at the indicated concentrations for 72 h, and then the proliferation of NSCLC cells was measured with a SRB assay. The mean combination index (CI) values are shown. (B and C) Colony formation assays were used to detect the proliferation of NCI-H460 cells. NCI-H460 cells were incubated with DYRK1A inhibitor, harmine, and/or Bcl-2 inhibitor (ABT-737 or ABT-263) at the indicated concentrations for 14 days, the cells were then stained with Giemsa solution, and the colony numbers were counted. Significant results are presented as *** *P* < 0.001.

Next, we hypothesized that DYRK1A inhibition would sensitize NSCLC cells to Bcl-2 inhibitors via suppression of Mcl-1. As expected, we observed synergistic cytotoxic effects when the DYRK1A inhibitor, harmine, was combined with either ABT-737 or ABT-199 in NSCLC cells, with mean CI values below 0.9 (**Figure 4A**). In contrast, treatment of cells with a combination of harmine and Bcl-2 antagonists significantly suppressed NCI-H460 cell colony formation compared to treatment with harmine alone or Bcl-2 antagonists alone (**Figure 4B and 4C**, combination therapy *vs.* monotherapy, *P* < 0.001). Thus, combined treatment with harmine and Bcl-2 antagonists dramatically reduced NSCLC cell proliferation.

**Figure 5:**
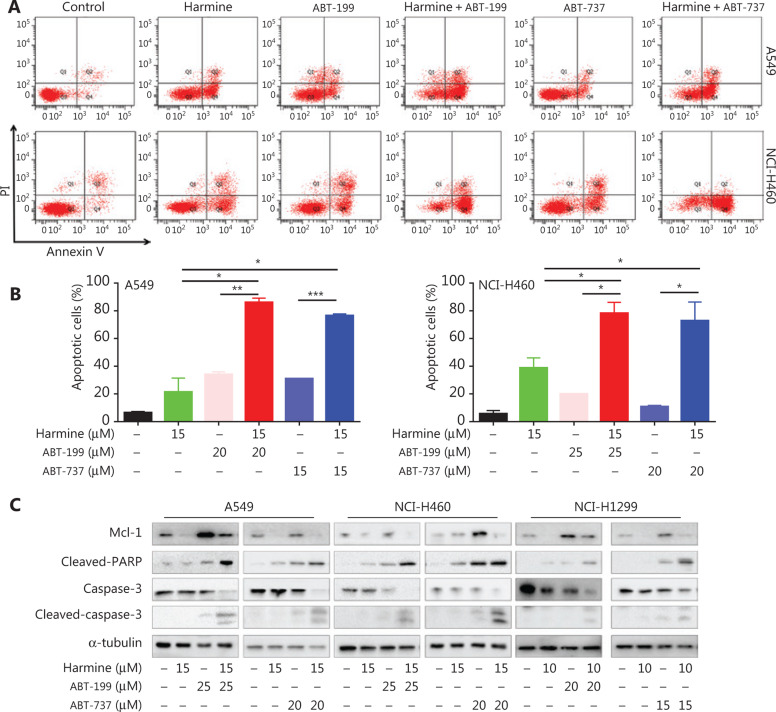
DYRK1A inhibitor, harmine, enhances the apoptosis induced by Bcl-2 inhibitors in NSCLC cells. (A and B) NSCLC cells were incubated with harmine and/or Bcl-2 inhibitor at the indicated concentrations for 48 h, and then the apoptosis was measured by Annexin V-FITC staining. (C) NSCLC cells were incubated with harmine and/or Bcl-2 inhibitor at the indicated concentrations for 48 h, and then Western blot was used to detect the expression of the indicated proteins. Significant results are presented as * *P* < 0.05, ** *P* < 0.01, or *** *P* < 0.001.

To determine whether harmine could enhance Bcl-2 antagonist-mediated apoptosis, we compared the effect of the Bcl-2 antagonist treatment with or without harmine on NSCLC cells. Harmine and Bcl-2 antagonists each elicited apoptosis as single agents, but a more profound effect was observed using combined treatment in NSCLC cells (**Figure 5A and 5B**, combination therapy *vs.* monotherapy, *P* < 0.05). Furthermore, PARP and caspase-3 showed greater activation with combined treatment compared to single treatment (**Figure 5C**). Treatment of NSCLC cells with Bcl-2 antagonist alone resulted in increased expression of Mcl-1, whereas Mcl-1 expression was markedly reduced following combined treatment with harmine and either ABT-737 or ABT-199. These data demonstrated that harmine enhanced Bcl-2 antagonist-mediated apoptosis in NSCLC cells by reducing Mcl-1 expression.

### DYRK1A inhibition sensitizes primary NSCLC cells to Bcl-2 inhibitors by reducing Mcl-1 expression

**Figure 6:**
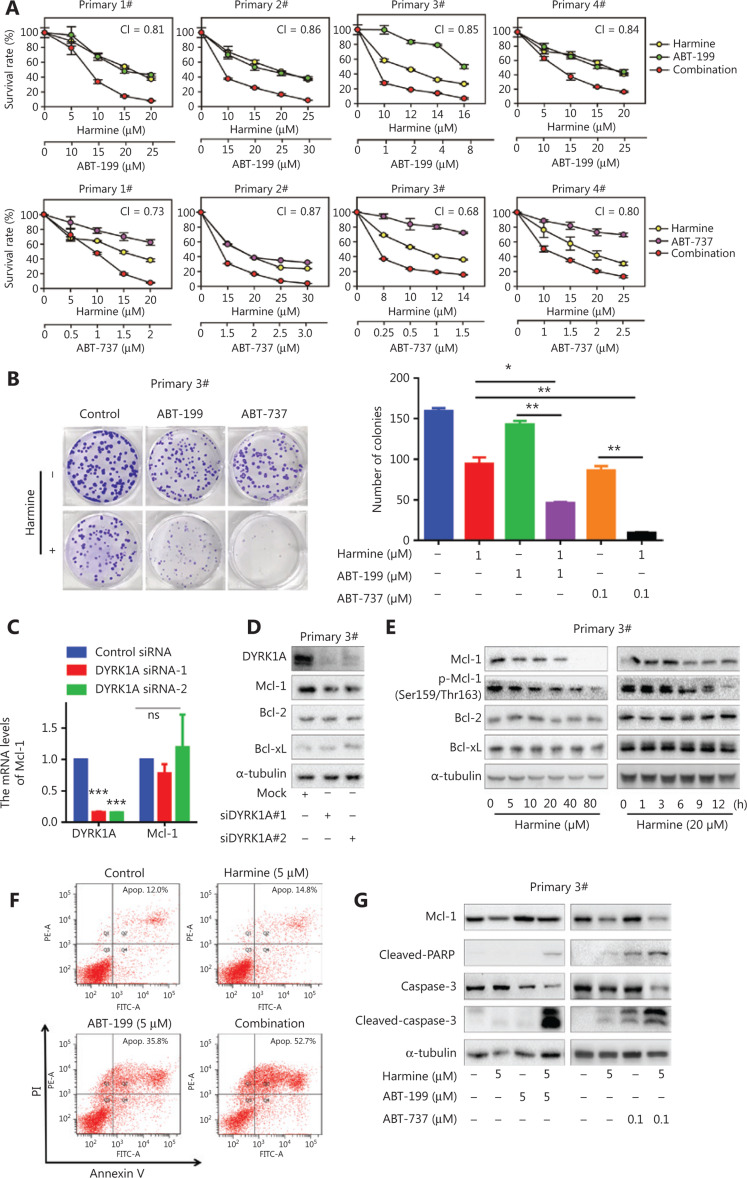
DYRK1A inhibition sensitizes primary NSCLC cells to Bcl-2 inhibitors *via* regulating Mcl-1. (A) Primary NSCLC cells were treated with harmine and/or Bcl-2 inhibitor at the indicated concentrations for 72 h, and then the proliferation of NSCLC cells was measured with a SRB assay. The mean combination index (CI) values are shown. (B) Primary NSCLC cells were incubated with harmine and/or Bcl-2 inhibitor at the indicated concentrations for 14 days, the cells were then stained with Giemsa solution, and the colony numbers were counted. (C) Primary NSCLC cells (#3) were transfected with control siRNA and DYRK1A siRNA for 48 h, and real-time reverse transcriptase PCR analyses were used to detect the mRNA levels of DYRK1A and Mcl-1. (D) Primary NSCLC cells (#3) were transfected with control siRNA and DYRK1A siRNA for 48 h, and the expression of DYRK1A and Bcl-2 family members were detected by Western blot. (E) Left panel: primary NSCLC cells (#3) were treated with harmine at the indicated concentration for 24 h, and the expression of the indicated proteins were detected by Western blot. Right panel: primary NSCLC cells (#3) were treated with 20 μM harmine for 1, 3, 6, 9, and 12 h, and the expression of the indicated proteins was detected by Western blot. (F) Primary NSCLC cells (#3) were incubated with harmine and/or ABT-199 at the indicated concentrations for 48 h, and then apoptosis was measured by Annexin V-FITC staining. (G) Primary NSCLC cells (#3) were incubated with harmine and/or Bcl-2 inhibitor at the indicated concentrations for 48 h, and then Western blot was used to detect the expression of the indicated proteins. Significant results are presented as * *P* < 0.05, ** *P* < 0.01.

Finally, to evaluate the clinical potential of combined harmine and Bcl-2 antagonist therapy, we established four primary NSCLC cancer cell lines from lung cancer patients. Combined treatment of primary NSCLC cell lines with harmine and Bcl-2 antagonist showed synergistic, anti-proliferative effects with the CI values below 0.9 (**Figure 6A**). Furthermore, combined treatment with harmine and Bcl-2 antagonist significantly suppressed primary NSCLC cell colony formation when compared to treatment with single agents (**Figure 6B**, combination therapy *vs.* monotherapy, *P* < 0.05). Although suppression of DYRK1A with siRNA had no effect on the level of Mcl-1 mRNA (**Figure 6C**), suppression of DYRK1A expression with siRNA or harmine reduced Mcl-1 protein expression in primary NSCLC cells (**Figure 6D and 6E**). In addition, harmine treatment enhanced Bcl-2 inhibitor-induced apoptosis in primary NSCLC cells by suppressing Mcl-1 expression (**Figure 6F and 6G**). Together, these data demonstrated that DYRK1A suppression enhanced the anti-NSCLC activity of Bcl-2 antagonists by reducing Mcl-1 expression, both in NSCLC cell lines and primary NSCLC cells.

## Discussion

Mcl-1 is an anti-apoptotic protein that plays a critical role in the development of resistance to anti-cancer drugs, including epidermal growth factor receptor-tyrosine kinase inhibitors (EGFR-TKIs) and Bcl-2 inhibitors^25^. Although direct Mcl-1 inhibitors are currently in preclinical and clinical development, none has been approved by the Food and Drug Administration. This highlights the need for alternative therapeutics that target the Mcl-1 pathway^26^. Mcl-1 is degraded in cancer cells after effective therapeutic treatment, and defective Mcl-1 degradation is correlated with intrinsic and acquired drug resistance^27^. In this study, we demonstrated that overexpression of DYRK1A in NSCLC cells significantly increased Mcl-1 expression, whereas suppression of DYRK1A inhibited Mcl-1 expression, indicating that DYRK1A is an upstream activator of Mcl-1 in NSCLC cells, including adenocarcinoma and squamous cell carcinoma (**Figure 1 and Supplementary Figure S1**). Furthermore, DYRK1A and Mcl-1 proteins showed co-localization in NSCLC cells and co-expressed in tumor samples from lung cancer patients, revealing that Mcl-1 may be a novel DYRK1A substrate. Therefore, we hypothesized that DYRK1A suppression would sensitize cancer cells to Bcl-2 inhibitor treatment by suppressing Mcl-1 expression. Indeed, treatment of NSCLC cells with harmine, a DYRK1A inhibitor, resulted in induction of apoptosis and suppression of cell proliferation following Bcl-2 inhibitor treatment. Furthermore, harmine enhanced the anti-NSCLC activity of Bcl-2 inhibitors in four primary NSCLC cell lines. In addition, our data showed that treatment of NSCLC cells with Bcl-2 inhibitor alone resulted in increased Mcl-1 expression, but combined treatment with harmine suppressed this increase in Mcl-1 expression. These data demonstrated that Mcl-1 was involved in the synergistic effect that was observed with combined harmine and Bcl-2 inhibitor treatment in NSCLC cells.

Phosphorylation of Mcl-1 at Thr-163 by extracellular signal-regulated kinase leads to an increase in the stability of Mcl-1 protein and contributes to the development of acquired resistance to Bcl-2 inhibitors^28,29^. Phosphorylation of Mcl-1 at Thr-163 also serves as a primer for subsequent glycogen synthase kinase-3 (GSK3)-mediated phosphorylation at Ser-159, which ultimately leads to Mcl-1 ubiquitination and subsequent proteasomal degradation^30–32^. We demonstrated that DYRK1A suppression by siRNA or harmine decreased the phosphorylation of Mcl-1 at Ser159/Thr163. DYRK1A suppression also accelerated Mcl-1 degradation, indicating that DYRK1A mediated proteasome-dependent degradation of Mcl-1. Furthermore, DYRK1A likely directly bound and phosphorylated Mcl-1, thereby enhancing Mcl-1 protein stability and contributing to the development of acquired resistance to Bcl-2 inhibitors. In this regard, our findings suggested that targeting of DYRK1A may serve as a novel approach to reduce Mcl-1 protein stability in NSCLC cells and thereby prevent Bcl-2 inhibitor resistance. However, further studies are required to identify the binding site between DYRK1A and Mcl-1.

NSCLC patients harboring EGFR-activating mutations derive greater benefit from EGFR-TKI therapy than from chemotherapy, either in first-line or subsequent lines of treatment^33^. However, the overall EGFR mutation rate is only 16.7% in NSCLC patients and most NSCLC patients harboring EGFR wild-type have poor responses to EGFR-TKI therapy. Thus, platinum-based chemotherapy is still the standard therapeutic regimen for these patients^34^. For these reasons, there is an urgent need to develop novel targeted therapeutic strategies for NSCLC patients with wild-type EGFR. Our previous work has demonstrated that DYRK1A positively regulates EGFR/Met via regulating the expression and activation of STAT3, and that suppression of DYRK1A enhances the anti-cancer activity of EGFR-TKIs in wild-type EGFR NSCLC cells via inhibiting the STAT3 signaling pathway^20^. Furthermore, inhibition of DYRK1A suppresses angiogenesis and tumor growth via the p53 signaling pathway in EGFR wild-type NSCLC cells^35^. These findings suggested that targeting of DYRK1A could be a novel, effective strategy for treatment of NSCLC patients with EGFR wild-type. In the present study, we demonstrated that combined treatment with a DYRK1A inhibitor and a Bcl-2 inhibitor produced a synergistic, anti-proliferative effect in EGFR wild-type NSCLC cell lines (NCI-H1299, A549, and NCI-H460) as well as in primary NSCLC cells (derived from three EGFR wild-type NSCLC patients and one patient with an EGFR exon 20 insertion mutation resistant to EGFR-TKI treatment; **Table 1**)^36^. Thus, this combined treatment regimen provides an attractive therapeutic strategy for the treatment of wild-type EGFR NSCLC patients.

## Conclusions

**Figure 7:**
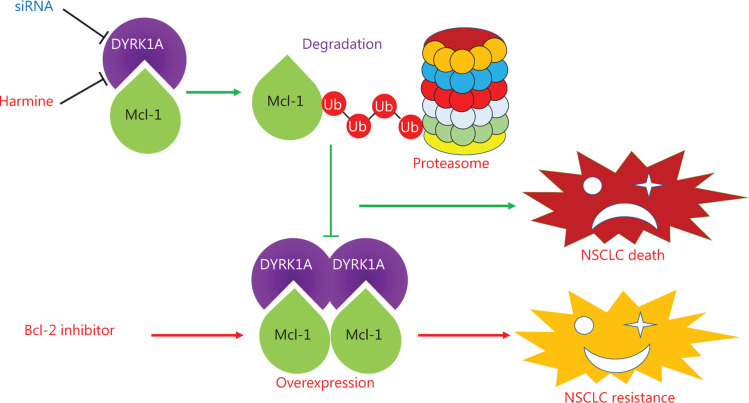
The summary of DYRK1A in regulating Mcl-1 and anti-NSCLC effects of Bcl-2 inhibitors. DYRK1A promotes the stability of Mcl-1, and genetic or pharmacological inhibition of DYRK1A promotes the proteasome-dependent degradation of Mcl-1 in NSCLC cells. Mcl-1 overexpression confers acquired resistance to Bcl-2 inhibitors in NSCLC. Thus, DYRK1A suppression sensitizes NSCLC cells to Bcl-2 inhibitors treatment via regulating Mcl-1.

In summary, we showed that either genetic or pharmacological inhibition of DYRK1A selectively perturbed the stability of Mcl-1. Furthermore, inhibition of DYRK1A enhanced the anti-NSCLC activity of Bcl-2 inhibitors by inhibiting Mcl-1 expression. Our data identified Mcl-1 as a novel substrate of DYRK1A and highlighted DYRK1A as a potential therapeutic target for conquering Bcl-2 inhibitor resistance in NSCLC (**Figure 7**).

## Figures and Tables

**Table 1:** Primary NSCLC patient information

Primary cells	Gender	Age (years)	Pathology	Immunohistochemistry	EGFR mutation
LS-1#	Male	58	Metastatic lung adenocarcinoma	TTF1[+], Napsin A[+], CK7[+], CK20[+], GFAP[&minus;], Ki-67[+,15%-20%]	EGFR (wt)
LS-2#	Female	52	Peripulmonary moderately to poorly differentiated lung adenocarcinoma	CK7[+], CK20[−], CK5/6[−], P63[−], Napsin A[+], TTF1[+], ALK[0], Ki-67[+,2%-5%]	EGFR (20 lns)
LS-3#	Male	71	Invasive lung adenocarcinoma	ROS1(−), c-Met(++,80%), CK5/6(−), CK7(+), P40(−), P63(−), TTF1(+), Napsin A(+)	EGFR (wt)
LS-4#	Male	71	Invasive lung adenocarcinoma	CK20(−), CDX-2(−), c-Met(+,10%), CK5/6(−), ROS1(+,5%), CK7(−), P40(−), P63(−), TTF1(+), Napsin A(+), ALK(D5F3)(−)	EGFR (wt)

EGFR, epidermal growth factor receptor; wt, wild-type.
