# Erratum to DYRK1A suppression restrains Mcl-1 expression and sensitizes NSCLC cells to Bcl-2 inhibitors

**DOI:** 10.20892/j.issn.2095-3941.2022.0172

**Published:** 2022-05-10

**Authors:** Yangling Li, Dongmei Zhou, Shuang Xu, Mingjun Rao, Zuoyan Zhang, Linwen Wu, Chong Zhang, Nengming Lin

**Affiliations:** 1 Department of Clinical Pharmacology, Hangzhou First People’s Hospital, Nanjing Medical University, Hangzhou 310006, China; 2 Department of Clinical Pharmacology, Key Laboratory of Clinical Cancer Pharmacology and Toxicology Research of Zhejiang Province, Hangzhou First People’s Hospital, Zhejiang University School of Medicine, Hangzhou 310006, China; 3 Institute of Pharmacology, College of Pharmaceutical Sciences, Zhejiang Chinese Medical University, Hangzhou 311402, China; 4 School of Medicine, Zhejiang University City College, Hangzhou 310015, China

In the published paper^1^, errors appeared in **Figure 1D** on page 391. In **Figure 1D**, an incorrect gel image was shown for Bcl-xL in NCI-H1299 cells. **Figure 1D** has been updated to correct the mistake above. The errors do not affect the conclusions of this article. We apologize for the errors and for any confusion that they may have caused.

**Figure 1:**
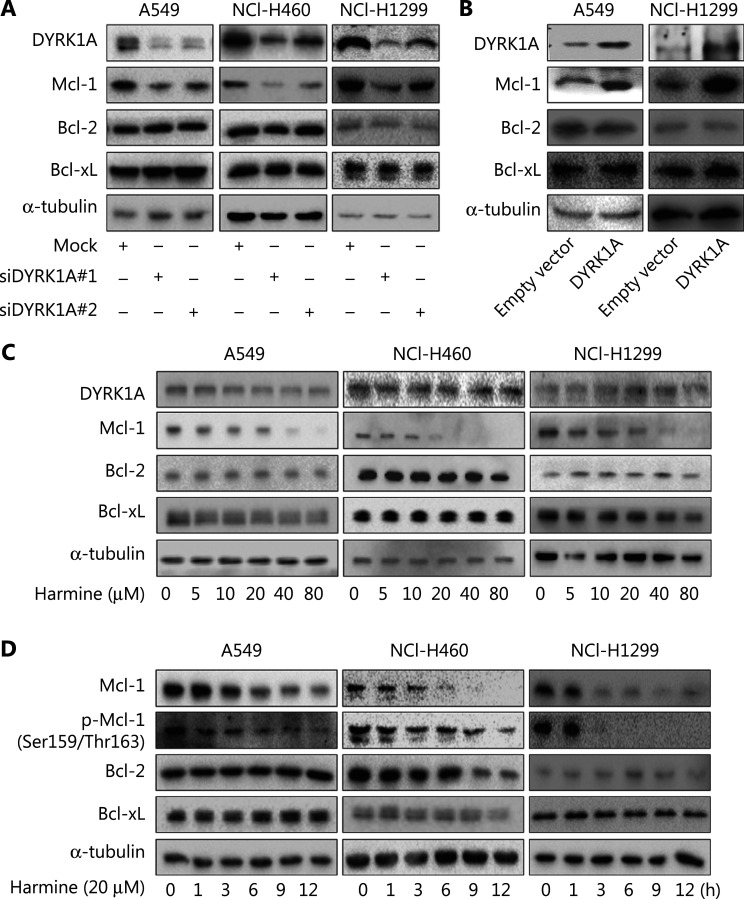
(C) NSCLC cells were treated with harmine at the indicated concentrations for 24 h, and the expression of the indicated proteins were detected by Western blot. (D) NSCLC cells were treated with 20 µM harmine for 1, 3, 6, 9, and 12 h, and the expression of the indicated proteins was detected by Western blot.
