# Supplementary material for: DYRK1A suppression restrains Mcl-1 expression and sensitizes NSCLC cells to Bcl-2 inhibitors
Source: Cancer Biol Med. 2020 May 15;17(2):387–400. doi: 10.20892/j.issn.2095-3941.2019.0380 (PMC7309455; doi:10.20892/j.issn.2095-3941.2019.0380)
Supplement: Supplementary file 1 [file cbm-17-387-s001.pdf]

Supplementary material

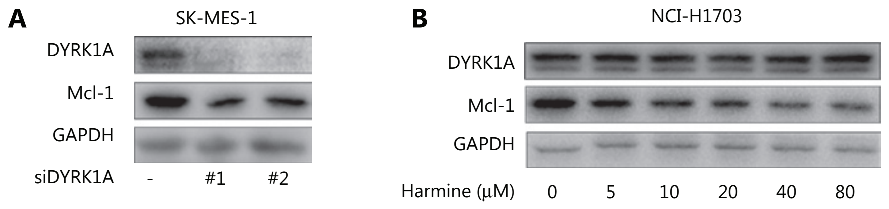

Figure S1 DYRK1A regulates the expression of Mcl-1 in squamous cell carcinoma cells. (A) SK-MES-1 cells were transfected with control siRNA and DYRK1A siRNA for 48 h, and the expression of DYRK1A and Mcl-1 were detected by Western blot. (B) NCI-H1703 cells were treated with harmine at the indicated concentrations for 24 h, and the expression of the indicated proteins was detected by Western blot.
